# The relative vertex clustering value - a new criterion for the fast discovery of functional modules in protein interaction networks

**DOI:** 10.1186/1471-2105-16-S4-S3

**Published:** 2015-02-23

**Authors:** Zina M Ibrahim, Alioune Ngom

**Affiliations:** 1 Institute of Psychiatry, King's College London, De Crespigny Park, SE5 8AF, London, UK; 2 NIHR Biomedical Research Centre for Mental Health and Biomedical Research Unit for Dementia, South London; 3 School of Computer Science, University of Windsor, 401 Sunset Avenue, N9B 3P4, Windsor, Ontario, Canada; 4 Maudsley NHS Foundation Trust, Denmark Hill, SE5 8AZ, London, UK

**Keywords:** Protein Complexes, Weighted Networks, Functional Modules, Network Clustering Criterion, Protein Interaction Networks

## Abstract

**Background:**

Cellular processes are known to be modular and are realized by groups of proteins implicated in common biological functions. Such groups of proteins are called *functional modules*, and many community detection methods have been devised for their discovery from protein interaction networks (PINs) data. In current agglomerative clustering approaches, vertices with just a very few neighbors are often classified as separate clusters, which does not make sense biologically. Also, a major limitation of agglomerative techniques is that their computational efficiency do not scale well to large PINs. Finally, PIN data obtained from large scale experiments generally contain many false positives, and this makes it hard for agglomerative clustering methods to find the correct clusters, since they are known to be sensitive to noisy data.

**Results:**

We propose a local similarity premetric, the *relative vertex clustering value*, as a new criterion allowing to decide when a node can be added to a given node's cluster and which addresses the above three issues. Based on this criterion, we introduce a novel and very fast agglomerative clustering technique, FAC-PIN, for discovering functional modules and protein complexes from a PIN data.

**Conclusions:**

Our proposed FAC-PIN algorithm is applied to nine PIN data from eight different species including the yeast PIN, and the identified functional modules are validated using Gene Ontology (GO) annotations from DAVID Bioinformatics Resources. Identified protein complexes are also validated using experimentally verified complexes. Computational results show that FAC-PIN can discover functional modules or protein complexes from PINs more accurately and more efficiently than HC-PIN and CNM, the current state-of-the-art approaches for clustering PINs in an agglomerative manner.

## Background

Functional modules are groups of genes or proteins involved in common elementary biological functions. Proteins are also known to interact with each other by forming complexes, and each such complex performs an independent and discrete biological function through the interactions of its member proteins [1]. Single proteins may also participate in more than one complex or functional module. Functional modules or protein complexes correspond to *modules*, which are dense subgraphs within protein interaction networks (PINs), and hence, can be discovered by appropriate network clustering approaches. Generally speaking, modules in PINs refer to highly connected sub-graphs which have more internal edges than external edges. Many definitions of modules have been proposed in literature [2], and consequently different community detection algorithms have been proposed based on these different definitions.

Module detection in PINs is a computationally hard task and conventional clustering algorithms are not well suited for this task [3,4]. Efficient, accurate, robust, and scalable methods are therefore required for mining large PINs [5-8]. There are generally three classes of modules detection approaches: 1) those based on finding *cliques*, which are fully connected sub-networks [9,10]; 2) those based on detecting dense subnetworks [11,12], not necessarily cliques; and 3) those based on uncovering the hierarchical organization of modules within PINs [13,14]. Clique techniques are not quite scalable to large PINs and the identified modules are too strict in the biological sense of modules since proteins participating in a complex may not all interact with each other. Current density-based algorithms commonly misclassify proteins with low degree into small clusters which could be merged to core protein clusters [15]. Moreover, many biologically meaningful modules are ignored due to their low topological connectivity [15].

Hierarchical clustering methods based on global metric over nodes or edges, such as betweenness centralities, are very time-consuming, and thus do not scale well to large PINs. The few hierarchical approaches based on local metric also have the common problem of classifying very low-degree vertices into separate clusters, which does not make sense biologically. Another major issue in current hierarchical clustering approaches is their inability to perform well on noisy data. This is generally the case when clustering PIN data generated from large scale high-throughput experiments. As discussed in [16,17], such PIN data usually contain many false positive interactions, and hence, care must be taken to deal with the sensitivity of hierarchical methods on such data.

The majority of the clustering methods proposed in the literature has focused on identifying nonoverlapping communities. However, it is well recognized that complex networks contain multi-class nodes corresponding to vertices belonging to many communities at once. Overlapping clustering algorithms have not been intensively studied nor successful at finding good subnetworks, although they first appeared three decades ago; see an extensive review of over-lapping methods in [18]. Multi-functional proteins are proteins which perform several functions and interact specifically with distinct sets of protein partners simultaneously or not, depending on the function being performed. Thus, such proteins are involved in many functional modules or protein complexes, and hence, it is reasonable to assume that PINs have overlapping communities, each containing some multi-functional proteins. Few successful hierarchical clustering approaches such as the *Overlapping Cluster Generator *(OCG) algorithm of [19] and the *Link Communities *method of [20] (to cite just a few) have been recently proposed with the aim of identifying overlapping protein communities as well as multi-functional proteins from PINs.

In this paper, we propose a fast agglomerative clustering technique, FAC-PIN, which addresses the issues and limitations discussed above for hierarchical algorithms. FAC-PIN is based on a local similarity pre-metric of *relative vertex-to-vertex clustering value *for clustering PINs in an agglomerative hierarchical manner.

### Related works

Many hierarchical clustering approaches (both agglomerative and divisive techniques) have been introduced in literature, since the original publication of [21] for clustering networks. See the excellent survey on graph clustering algorithms in [22]. Thus, we will present only the few methods that are directly related to our proposed agglomerative approach.

An effective agglomerative technique for clustering large networks was first proposed by [21]. The Girvan-Newman (GN) algorithm [21] first computes the *edge-betweenness *centrality value of each edge; this is a global metric over the edges and is defined as the number of shortest paths containing a given edge. Then, GN subsequently sort and then remove edges with large betweenness values in an iterative manner and in order to detect the communities; since such edges correspond to *bridges *connecting two modules whereas low-betweenness edges are internal to modules. To increase the computational speed of GN, [23] made a simple but non-trivial modification in the computation of the value of the modularity function used in GN. [15] defined the concept of the degree of a subnetwork *S *as the number the of edges containing one endpoint inside *S *and the other endpoint outside *S*. The degree of subnetworks was used along with the edge-betweenness values to devise an agglomerative method for module discovery. [14] developed a fast agglomerative approach for community detection based on a global centrality measure, the *vertex clustering coefficient *; which is defined as the ratio of the number of edges between the neighbors of a given vertex *v *and the total number of possible edges in that neighborhood, it measures the degree of completeness of the subnetwork defined by *v *and its neighbors [24]. [2] designed an agglomerative technique based on the clustering coefficient of an edge; the *edge clustering coefficient *extends the vertex clustering coefficient and is a global measure defined as the number of triangles to which a given edge *e *= (*u, v*) belongs to, divided by the number of triangles that might potentially include (*u, v*). That is:

(1) $$C_{u,v}^{\left(3\right)}=\frac{Z_{u,v}^{\left(3\right)}}{\text{min}\left\{\left(k_{u}-1\right)\left(k_{v}-1\right)\right\}},$$

where, *k_a _*is the degree of a vertex *a*, $Z_{u,v}^{\left(3\right)}$ is the number of triangles containing edge (*u, v*), and min{(*k_u _*- 1), (*k_v _*- 1)} is the maximal possible number of triangles containing (*u, v*). This coefficient has been further generalized to higher-order cycles, $C_{u,v}^{\left(k\right)}$, such as squares for *k *= 4, $C_{u,v}^{\left(4\right)}$. Edges contained in few or no triangles have low clustering coefficients, and hence, correspond to *bridges *connecting two clusters. The edge clustering coefficient assumes the existence of cycles of length *k *in a network; which is problematic since a network can have many cycles of different lengths and the length distribution is unknown (e.g., there may be very few or very many short-length cycles). For this reason, [25] defined a local node similarity metric over the edges, the *edge clustering value*, which is not based on cycles but on the common neighbors of the two endpoints of edge (u; v). The edge clustering value is defined as:

(2) $$ECV\left(u,v\right)=\frac{|N_{u}\cap N_{v}{|}^{2}}{\left|N_{u}|\cdot |N_{v}\right|},$$

where, *N_a _*is the set of neighbors of a vertex *a *and its cardinality is defined as |*N_a_*|. Here, endpoints vertices of an edge (*u, v*) with a larger clustering value are more likely to be in the same cluster. Using the edge clustering value, [25] devised an agglomerative technique, the HC-PIN algorithm, for discovering modules of a PIN and which is faster and more accurate than current hierarchical algorithms for network clustering. The edge clustering objectives in Equations (1) and (2) do not take into account the reliability of interactions in the presence of false positives in PIN data, and hence, will yield incorrect clustering results. In this regards, [25] modified the objective of Equation (2) to account for noise in the PIN data, as

(3) $$ECV_{w}\left(u,v\right)=\frac{\sum_{k\in I_{u,v}}w\left(u,k\right)\cdot \sum_{k\in I_{u,v}}w\left(v,k\right)}{\sum_{s\in N_{u}}w\left(u,s\right)\cdot \sum_{t\in N_{v}}w\left(v,t\right)},$$

where *I_u,v _*= *N_u _*∩ *N_v _*, and 0 ≤ *w*(*a, b*) ≤ 1 is the weight assigned to the edge (*a, b*) and which represents the reliability of the interaction between vertices *a *and *b *or the probability of their interaction being a true positive. Clearly, Equation (2) is a special case of Equation (3) for weighted undirected graph with *w*(*a, b*) = 1 for all edges (*a, b*). In Equations (1)-(3), two vertices connected by an edge with larger objective value are more likely to lie in the same module.

Recently, while finalizing this manuscript, we have been made aware of an hierarchical approach introduced in [20] and which focuses on grouping links (i.e., edges) rather than vertices, in contrast to the existing literature which has almost entirely focused on grouping nodes. It is well-know that communities in complex networks often overlap such that nodes simultaneously belong to several groups at once, which in turn, are known to be involved into hierarchical structures. It has therefore proved difficult for node-focused community detection methods to accurately identify relevant functional modules because of the hierarchical structures of the overlapping groups. Let $N_{a}^{+}$ denotes the set of node *a *and its neighbors and *e_a,b _*denote the edge (*a, b*), then by defining network communities as groups of links rather than groups of vertices, [20] proposed the following similarity function for link pairs that share a node in an undirected unweighted network

(4) $$S\left(e_{u,k},e_{v,k}\right)=\frac{\left|N_{u}^{+}\cap N_{v}^{+}\right|}{\left|N_{u}^{+}\cup N_{v}^{+}\right|},$$

and applied a simple single-linkage hierarchical clustering algorithm to build an link dendrogram from Equation (4) which yields link communities with the best edge partition density. By identifying such non-overlapping link communities, [20] has detected hierarchically organized node community structures with pervasive overlap.

In the next section, we will propose a new criterion for weighted undirected graphs, which is a modification of the *relative vertex-to-vertex clustering value *which we have first introduced in [26] for un-weighted graph; in [26], however, the unweighted criterion was applied only to the problem of detecting protein complexes in PINs [27] whereas here we apply our weighted criterion here for identifying functional modules in PINs. It is a local similarity premetric combining the ideas behind the vertex clustering coefficient, the edge clustering coefficient, and the edge clustering value, and which allows to decide when a given vertex can be included into the cluster of another vertex, and which helps address all of the issues discussed above.

## Methods

### Network modularity structure

The concept of community is qualitative rather than quantitative; that is, nodes must be more densely connected within the community than with the rest of the network. The quantitative definition of the modularity of a network is still an open debate. Here, we use the modularity quality function *Q *which was introduced by the authors of [28], and which is a widely used quantitative measure for evaluating the modular structure of a network. Specifically, given an un-weighted undirected graph *G *= (*V, E*) with |*V*| = *n*, its symmetric adjacency matrix *A *= [*A_u,v_*]_*n *× *n *_where *A_u,v _*= 1 if nodes *u *and *v *are connected and otherwise *A_u,v _*= 0. Then, the modularity *Q *function is defined as

(5) $$Q\left(P_{k}\right)=\overset{k}{\underset{i=1}{\sum }}\left(e_{ii}-a_{i}^{2}\right),$$

where: *P*(*k*) = ({*C*_1_,...,*C_k _*}) is a partition of *V *into *k *groups; $e_{ii}=\frac{L\left(C_{i},C_{i}\right)}{L\left(V,V\right)}$ is the fraction of edges with both end vertices in the same community *i*; $a_{i}=\frac{L\left(C_{i},V\right)}{L\left(V,V\right)}$ is the fraction of edges with at least one end vertex in community *C_i_*; and, $L\left(S_{1},S_{2}\right)=\sum_{u\in S_{1},v\in S_{2}}A_{u,v}$. Larger values of *Q *correspond to more distinct community structures in PINs. Function *Q *have serious *resolution limits *which have been discussed at length in [22], and the size of a detected community depends on the size of the whole network; thus, the choice of partition is highly sensitive to the total number of edges in the network. A second partition scoring function Ω which seeks to improve *Q *has been introduced in [29] and is defined as

(6) $$\Omega \left(P_{k}\right)=\overset{k}{\underset{i=1}{\sum }}\left(e_{ii}\cdot \text{log}a_{i}\right).$$

Function Ω allows for more diverse cluster sizes than function *Q *and which are not too small and not too large, and smaller values corresponds to better modularity structures. A third scoring function, the *modularity density *function *D *of [14], overcomes the resolution limits of *Q *by directly including information on the number of nodes in a community. It is defined as

(7) $$D\left(P_{k}\right)=\overset{k}{\underset{i=1}{\sum }}\frac{L\left(C_{i},C_{i}\right)-L\left(C_{i},{\bar{C}}_{i}\right)}{\left|C_{i}\right|},$$

where, ${\bar{C}}_{i}=V\backslash C_{i}$ is the set of vertices not in *C_i_*. Thus, the aim of function *D *is to optimize both the modularity and the density of a community. For weighted undirected graphs *G *= (*V, E*) with weights assigned to edges in *E*, we propose new modularity functions, *Q_w_*, Ω*_w _*and *D_w_*. These three functions are direct generalizations of *Q*, Ω and *D *above, with *L*(*S*_1_, *S*_2_) redefined for weighted undirected graphs as

(8) $$L\left(S_{1},S_{2}\right)=\underset{u\in S_{1},v\in S_{2}}{\sum }w\left(u,v\right).$$

The problem of community detection is hence equivalent to searching for a *k *and a partition *Pk *to maximize the value of a modularity function.

### The relative vertex-to-vertex clustering value

Suppose an edge (*u, v*) in a scale-free network such that *u *has lower degree than *v*. We can reasonably *assume that u has more likely joined the cluster containing v than v has joined the cluster containing u*. This assumption stems from the principle of *preferential attachment *in power-law networks, which states that a new node *u *is likely to *attach *to a high-degree node *v *than to a low degree node. The *edge clustering coefficient *$C_{u,v}^{\left(k\right)}$ of [2] and the *edge clustering value ECV *(*u, v*) of [25] are similarity metrics which treat both endpoints of edges (*u, v*) equally, irrespective of their degrees. Also, another issue is that both *ECV *(*u, v*) and $C_{u,v}^{\left(3\right)}$ require vertices *u *and *v *to be connected by an edge. This requirement is quite restrictive and we aim to extend (in the future) to the case in which pair (*u, v*) is not an edge while still being able to decide if both vertices are in the same cluster. Finally, hierarchical approaches based on *ECV *(*u, v*) and $C_{u,v}^{\left(3\right)}$, or other objective functions, have the common problem of classifying low-degree vertices (peripheral to dense subnetwork modules) into separate clusters rather than merging them with their neighboring modules. These criteria tell how likely that *both u *and *v *lie in the same cluster, and not which of *u *or *v *has likely joined the other's cluster. Let *N_a _*be the set of neighbors of a vertex *a *in an un-weighted undirected graph *G *= (*V, E*). We define $N_{a}^{+}=N_{a}\cup \left\{a\right\}$ as the neighbor set of *a *augmented with *a *itself. Given two vertices *u *and *v*, we define the *clustering value of u relative to v *as:

(9) $$R\left(u--\to v\right)=\frac{\left|N_{u}^{+}\cap N_{v}^{+}\right|}{\left|N_{u}^{+}\right|}$$

To consider the reliability of edges in the presence of false positive interactions in the the PIN data, we modify the objective of Equation (9) to apply for weighted graphs, as follows

(10) $$R_{w}\left(u--\to v\right)=\frac{\sum_{a\in I_{u,v}^{+};\left(u,a\right)\in E;\left(a,v\right)\in E}w\left(u,a\right)\cdot w\left(a,v\right)}{\sum_{b\in N_{u}^{+};\left(u,b\right)\in E}w\left(u,b\right)},$$

where, $I_{u,v}^{+}=N_{u}^{+}\cap N_{v}^{+}$, and 0 ≤ *w*(*x, y*) ≤ 1 is the weight assigned to the edge (*x, y*) and which represents the reliability of the interaction between vertices *a *and *b *or the probability of their interaction being a true positive. Clearly, Equation (9) is a special case of Equation (10) for weighted undirected graph with *w*(*x, y*) = 1 for all edges (*x, y*). For a node *a *∈ *V*, we let $k_{a}=\sum_{b\in V}A_{a,b}$ be its degree. For a weighted graph, we define the *weighted degree *of a vertex *a *as $\kappa_{a}=\sum_{b\in V}w\left(a,b\right)$, similarly to [25].

$R_{w}(u--\to v)$, with 0 ≤ $R_{w}(u--\to v)$ ≤ 1, is a similarity premetric since it does not satisfy the axiom of symmetry and the triangle inequality but satisfies the axioms of self-similarity and maximality [30]; see http://www.scholarpedia.org/article/Similarity_measures and http://en.wikipedia.org/wiki/Metric_(mathematics)#Premetrics. A vertex *u *with a larger clustering value given another vertex *v *is more likely to lie in the cluster containing *v*. In the following we let *C*(*v*) = (*C_v _*⊂ *V, E_v _*⊂ *E*) denotes the subnetwork cluster containing *v *and we assume *C*(*v*) is a community. Below, we describe the properties of $R_{w}(u--\to v)$.

### Analysis of $R_{w}(u--\to v)$

In the following, we limit our discussions to the case of un-weighted networks, though they also apply to weighted networks. To understand how the similarity premetric $R_{w}(u--\to v)$ can be used to determine the communities in a network, we now discuss the relationships between values R(u--→v) and R(v--→u), and all the four possible cases of connectivity of an edge (*u, v*). The main question we address below is: when should we merge the vertex *u *with the current cluster *C*(*v*) of *v*?

1 Case *k_u _*= 1. R(u--→v) = 1, thus it is maximal. R(u--→v) is also maximal when *kv *= 1, and hence, the connected component *C *= ({*u, v*}, (*u, v*)) is a community. If on the other hand *k_v _*> 1, then we have R(u--→v) >R(v--→u) and therefore *u *should be merged with the current cluster *C*(*v*) of *v *(not the other way around, which corresponds to merging *v *with *C*(*u*)).

2 Case 1 <*k_u _*<*k_v_*. R(u--→v) >R(v--→u) and R(u--→v) may or may not be maximal. Vertex *u *should be merged with *C*(*v*) only when R(u--→v) > 0.5; that is, when more than 50% of the neighbors of *u*, $N_{u}^{+}$, are in the intersection, $N_{u}^{+}\cap N_{v}^{+}$. This is a reasonable decision since the number of triangles involving the edge (*u, v*) is |*N_u _*∩ *N_v_*|, and that the edge (*u, v*) is definitely not a "bridge" connecting two clusters when most of *u*'s neighbors form a triangle with *v*.

3 Case 1 <*k_v _*<*k_u_*. This is the reverse of case 2 above: thus, *u *should not merge with *C*(*v*) since R(u--→v) <R(v--→u).

4 Case *k_u _*= *k_v_*. R(u--→v) = R(u--→v), and we should consider two possible sub-cases.

(a) Sub-case $N_{u}^{+}=N_{v}^{+}$. We have R(u--→v) = R(u--→v) = 1 since $N_{u}^{+}=N_{v}^{+}=N_{u}^{+}\cap N_{v}^{+}$. Hence, *u *should be merged with *C*(*v*) given that the induced subnetwork of *G *for $N_{u}^{+}\cap N_{v}^{+}$ forms a community.

(b) Sub-case $N_{u}^{+}\neq N_{v}^{+}$. We have R(u--→v) = R(v--→u) < 1. In this case, *u *should be merged with *C*(*v*), only when R(u--→v) > 0.5.

Given an edge (*u, v*), assume the degrees of vertices *u *and *v *in *G *are such that *k_u _*= *k_v _*= *d *are (very) large and that *u *and *v *do not have common neighbors. Then, we have $R\left(u--\to v\right)=R\left(v--\to u\right)=1\cdot \frac{2}{1+d}\leq 0.5$ assuming *d *≥ 3. In this case, the induced subnetwork of *G *for {*u*} ∪ *C_v _*(or for $N_{v}^{+}$) is not a community, and likewise for {*v*} ∪ *C_u _*(or for $N_{u}^{+}$). In general, consider the induced subgraph of *G *on $N_{u}^{+}\cup N_{v}^{+}$ we define the *local betweenness value *of edge (*u, v*) as the percentage of paths from vertices in *N_u _*\ *N_v _*to vertices in *N_v _*\ *N_u _*going through edge (*u, v*). Given the number of common neighbors between *u *and *v*, |*N_u _*∩ *N_v_*|, the local betweenness of edge (*u, v*) is thus $\lambda \left(u,v\right)=100\cdot \frac{1}{|N_{u}\cap N_{v}|+1}$. Given two connected high-degree vertices *u *and *v*, the local edge betweenness value *λ*(*u, v*) increases as |*N_u _*∩ *N_v_*| decreases, and hence, it corresponds to when both R(u--→v) and R(v--→u) values are both small (and both ≤ 0.5) at the same time. Edges with high local betweenness values are edges which are *likely *connecting two communities, and therefore, vertices *u *and *v *should not lie in the same community. This is not necessarily true since we are making an inference based not on the *global edge betweenness *metric defined in [21]. However, starting with correct initializations and using an appropriate node clustering mechanism, a greedy algorithm can be devised based on the faster local evaluations instead of the costly global evaluations.

R(u--→v) is maximal when $\left|N_{u}^{+}|=|N_{u}^{+}\cap N_{v}^{+}\right|$; that is either Case (1) or Case (4a) above. In either cases, *u *contributes only new internal edges in the induced subnetwork of *G *for $C_{v}^{+}=\left\{u\right\}\cup C_{v}$ (or for $C_{v}^{+}=N_{v}^{+}$) and contributes no new external edges, and hence, the induced subnetwork of *G *for $C_{v}^{+}$ remains a community if *C_v _*(or $N_{v}^{+}$) is a community. Finally, *u *is more likely to be in the community *C*(*v*) and *v *less likely to be in the community *C*(*u*) when both R(u--→v) > 0.5 and R(u--→v) ≥ R(v--→u). Since R(u--→v) > 0.5 then *k_u _*≤ *k_v _*and $|N_{u}^{+}\cap N_{v}^{+}|=\frac{\left|N_{u}^{+}\right|}{2}$; that is, more than 50% of the neighbors of *u *are in the intersection and less than 50% of the neighbors of *v *are in the intersection. Since *k_u _*≤ *k_v _*then clearly the induced subnetwork of *G *for $C_{v}^{+}=\left\{u\right\}\cup C_{v}$ is a community when $N_{u}\cap N_{v}\subseteq C\left(v\right)$ with its modularity increasing with |*N_u _*∩ *N_v_*|.

### Quantitative definition of module

Given the four cases above and a user-defined merging parameter *μ *with 0 ≤ *μ *< 2, the decision to merge a node *u *with the cluster *C*(*v*) of a node *v *can be summarized into a single test containing all the four cases; that is: include *u *to *C*(*v*) whenever

$$R_{w}\left(u--\to v\right)>0.5\mu \;\text{and}\;R_{w}\left(u--\to v\right)\geq R_{w}\left(v--\to u\right).$$

The communities (i.e. modules) *C *determined by algorithms which use this merging test are such that the merging condition is satisfied for every internal edge of *C *and not satisfied for every external edge of *C*. Given a weighted undirected graph *G *= (*V, E*) and the merging parameter *μ*, a subgraph *C *⊆ *G *is said to be a *μ*-module if if the the condition for merging is true for every internal edge of *c *and false for every external edge of *C*. Different networks modularity structures are obtained by varying the value the merging parameter *μ*.

The relative vertex clustering value, R(u--→v) implements the ideas behind the edge clustering coefficient, $C_{u,v}^{\left(k\right)}$, of [2], since for a given vertex *v *and a neighbor *u *the number of triangles given edge (*u, v*) is exactly |*N_u _*∩ *N_v_*|; and *u *will be included into *C*(*v*) whenever most of the neighbors of *u *(excluding *v*) are in *N_u _*∩ *N_v _*. This is also true even when (*u, v*) is not an edge; in such case, |*N_u _*∩ *N_v_*| relates to the number of squares containing vertices *u *and *v*. On the other hand, we break through the limitations of [2] as in the edge clustering value, *ECV *(*u, v*) of [25], by not assuming the existence of closed loops in a networks, such as triangles or high-order loops. The relative vertex clustering values R(u--→v) and $R_{w}(u--\to v)$ also improves *ECV *(*u, v*) and *ECV w *(*u, v*) since neighbors *u *of *v *which have most of their neighbors forming a triangle with *v *are considered for possible inclusion in *C*(*v*). Searching for vertices *u *which form a cluster with *v *is also more efficient than searching for edges (*u, v*) that make a cluster since the number of edges is larger than the number of vertices in dense subgraphs.

### The FAC-PIN algorithm

In a clustering task, we can use $R_{w}(u--\to v)$ and $R_{w}(v--\to u)$ to decide whether *u *should be included into *C*(*v*) = (*C_v_, E_v_*) ⊂ *G *= (*V, E*), the current cluster of *v*. Based on the definitions of relative vertex-to-vertex clustering value and quantitative network modularity, we propose a fast agglomerative clustering node-focused algorithm named FAC-PIN, shown in Algorithm 1. The input to algorithm FAC-PIN is an undirected weighted graph; when un-weighted graph is used, then all edges (*a, b*) are treated equally with weight *w*(*a, b*) = 1. The output of FAC-PIN is a collection of non-overlapping subnetwork communities.

Given a weighted undirected PIN *G *= (*V, E*), we initially consider each vertex as a singleton cluster, and sort the vertices *v *∈ *V *into a queue *Q_V _*in non-increasing order of their weighted degrees *κ_v_*. Then,

**Algorithm 1 **The *FAC-PIN *algorithm

**Require: ***G *= (*V, E*): undirected PIN graph;

      *A*_|*V*| × |*V*|_: adjacency matrix;

      *W*_|*V*| × |*V*|_: weight matrix;

      *μ*: merging parameter;

**Ensure: ***P_k _*= {*C*_1 _,..., *C_k_*}: non-overlapping subnetwork communities

{**Initialization Phase**}

**for all ***v *∈ *V ***do**

      *C_v _*← {*v*}; {*C_v _*= **cluster ***containing node v*}

      *E_v _*← ∅;

      $\kappa_{v}\leftarrow \sum_{b\in V}w\left(v,b\right)$; {*weighted degree of v*}

      *C*(*v*) ← (*C_v_, E_v_*); {*Each vertex is a singleton cluster *}

                                 {*C*(*v*) = **subnetwork ***containing node v*}

end for

{**Community Detection Phase**}

Sort *V *to *Q_V _*in non-increasing order of *κ_v _*values;

repeat

   *v *← *Q_V_*; {*Select highest κ_v _vertex in Q_V_*}

   *N_v _*← {*u *∈ *V*| (*u, v*) ∈ *E*}; {*Neighbor set of v*}

   **for all ***u *∈ *N_v _*not yet assigned to a cluster **do**

      **if **$R_{w}(u--\to v)$ > 0.5*μ ***and **$R_{w}(u--\to v)$ ≥ $R_{w}(v--\to u)$

      **then**

         *C_z _*← *C_v _*∪ {*u*}, ∀ ∈ *C_v _*∪ {*u*};

      **end if**

   **end for**

   *Q_V _*← *Q_V _*- *v*; {*Remove v *from *Q_V_*}

**until ***Q_V _*= ∅

{**Compute the Partition ***P_k_*}

*U *← *V*;

*i *← 1;

**while ***U *≠ ∅ **do**

   *v *← randomly select a vertex from *U *;

   *C_i _*← *C*(*v*) = the induced subgraph of *G *for *C_v _*;

   *U *← *U*\{*u*|*C_u _*= *C_v_*};

   *i *← *i *+ 1;

end while

**return ***P_k _*← {*C*_1_,...,*C_k_*}; *Q_w _*(*P_k_*) and Ω*_w _*(*P_k_*);

{**Evaluate the Modularity of Partition ***P_k_*}

*Modularity *← *D_w_*(*P_k_*), *Q_w_*(*P_k_*) and Ω*_w_*(*P_k_*);

in an iterative manner, we select the next highest *κ_v _*vertex *v *from *Q_V _*and then we iteratively apply the merging condition

$$R_{w}\left(u--\to v\right)>0.5\mu \;\text{and}\;R_{w}\left(u--\to v\right)\geq R_{w}\left(v--\to u\right)$$

on each neighbor *u *∈ *N_v _*of *v *in order to decide for its inclusion into the current cluster *C_v _*of *v*.

A neighbor *u *∈ *N_v _*is added into the current cluster *C_v _*of *v*, when the majority of the neighbors of *u *are in $N_{u}^{+}\cap N_{v}^{+}$. That is when, R(u--→v) > 0.5 and $R_{w}(u--\to v)$ ≥ $R_{w}(v--\to u)$; in which case *κ_u _*≤ *κ_v _*and $\left|N_{u}^{+}\cap N_{v}^{+}|>\frac{1}{2}|N_{u}^{+}\right|$ which for weighted graphs is equivalent to $\sum_{a\in I_{u,v}^{+}}w\left(u,a\right)>\frac{1}{2}\sum_{b\in N_{u}^{+}}w\left(u,b\right)$ where $I_{u,v}^{+}=N_{u}^{+}\cap N_{v}^{+}$. By gradually examining each high-degree vertex *v *from the queue *Q_V _*and then gradually adding its un-assigned neighbors *u *to *C_v_*, FAC-PIN agglomerates all singleton clusters into |*V*| vertex sets *C_v_*. The final *k *communities *C_i_*, for 1 ≤ *i *≤ *k*, are the induced subgraphs of *G *for all *distinct C_v_*; in the algorithm, we made a distinction between a cluster *C_v _*= {*v*_1_,...,*v_n_*}, a subnetwork *C*(*v*) = (*C_v_, E_v_*), and the *i*-th subnetwork *C*_*i*_. In FAC-PIN, the merging parameter *μ *with 0 ≤ *μ *< 2 is user-defined. In particular for weighted PINs, different modularity results can be obtained by changing the values of *μ*

Most hierarchical methods, with the exception of the HC-PIN algorithm of [25], are based on a costly global metric for partitioning a PIN network. FAC-PIN is based on the local similarity premetric $R_{w}(u--\to v)$, which encodes useful information about the local topology around vertices *u *and *v*, and which helps make a local decision maximizing the modularity of the final partitioning.

#### Computational complexity of FAC-PIN

Given weighted PIN *G *= (*V, E*), let *n *= |*V*|, *m *= |*E*|, *κ*_max _= max_*v*∈*V *_*κ_v _*be the maximum weighted degree in *G*, and $\kappa_{ave}=\frac{1}{n}\sum_{v\in V}\kappa_{v}$ be the average weighted degree in *G*. The complexity of computing $R_{w}(u--\to v)$ is *O*(*κ*_max_), and hence, the complexity of FAC-PIN is $O\left(n\kappa_{ave}^{2}\right)\ll O\left(n\kappa_{\text{max}}^{2}\right)⋘O\left(n^{3}\right)$. PINs are power-law networks, thus the majority of proteins interact with few proteins only, and thus *κ_ave _*is generally small and can be considered a constant [25]. The CNM [23] and the HC-PIN [25] methods run in *O*(*mh *log *n*) and $O\left(m\kappa_{ave}^{2}\right)$ steps, respectively; where, *h *is the depth of the *dendrogram *describing the network's community structure. These are the currently fastest agglomerative methods. The space complexity of the three algorithms is *O*(*m*^2^). The main achievement with respect to computational complexity is that the cost of FAC-PIN is dependent on the number of nodes, rather than the number of edges, specially when *κ_ave _*is regarded as a constant in scale-free networks.

## Results and discussion

We have carried out several computational experiments on nine PIN data from eight different species using our proposed FAC-PIN algorithm. In this section, the data sets and the evaluation methods used in our experiments are described first. Next, we discuss the effect of varying the merging parameter *μ *on the FAC-PIN clustering results. Then, we arbitrarily set the merging parameter to *μ *= 0.5 and then proceed to compare and study the clustering results of the FAC-PIN approach with those of the HC-PIN and CNM methods on the same PIN data sets; the three algorithms are compared on (i) the functional enrichment of their predicted modules, (ii) their sensitivity, specificity, and *F *-score, (iii) the network modularity structure of the partitioning results, and finally, (iv) their execution times.

All computational experiments were performed on an Intel machine (Core TM i5-1600, 2.400 GHz, CPU with 8 GB RAM). The program codes were all written in R.

### PIN data sets

Original un-weighted PIN data of eight distinct species was downloaded from the REACTOME database http://www.reactome.org/download/all_interaction.html and one species from the DIP database [31]. The eight PIN data from REACTOME are listed here along with their number of proteins and interactions in parenthesis are: *B. taurus *(5737, 113888), *T. guttata *(Finch bird, 3929, 74314), *X. tropicalis *(Frog, 5473, 122706), *H. sapiens *(Human, 8997, 34935), *O. sativa *(Rice, 3778, 320570), *S. scrofa *(Wild boar, 5303, 119920), *D. rario *(Zebra fish, 8188, 274358), and *S. cerevisiae-1 *(Baker's yeast, 5697, 50675). The PIN data from DIP is *S. cerevisiae-2 *(Baker's yeast, 4726, 15166). In all these PIN data, the number of edges is much larger than the number of vertices.

We also downloaded a list of protein complexes obtained from the MIPS database, which we consider as a *gold standard *data. We extracted the protein complexes corresponding to the *S. cerevisiae-2 *PIN data from the MIPS *Comprehensive Yeast Genome Database-CYGD *ftp://ftpmips.gsf.de/fungi/yeast/catalogues/complexcat/complexcat_data_18052006. We proceeded similarly to [29] and considered only the known complexes (i.e., not those obtained by computational means) containing at least three proteins. Since FAC-PIN generates non-overlapping clusters, we considered only known complexes which are at the bottom of the MIPS hierarchy of complexes and subcomplexes. The unconfirmed complexes, that is those in category 550, were excluded.

### Evaluation methods

In order to study and compare the performance of FAC-PIN, we downloaded the CNM code http://cs.unm.edu/~aaron/research/fastmodularity.htm[23] and implemented the HC-PIN algorithm [25]. The two methods were applied on the same PIN data as FAC-PIN. For HC-PIN, we set the two parameters *λ *and *s *as in [25]; CNM has no parameters. Of the three algorithms, only FAC-PIN and HC-PIN can cluster weighted PINs. There are other network clustering approaches which we could compare FAC-PIN with, however they are either not designed for clustering weighted PINs or they are not hierarchical agglomerative algorithms. It should be noted that [25] compared his HC-PIN algorithms with six others PIN clustering approaches on the same *S. cerevisiae-2 *PIN data; none of them are hierarchical and only three of them can cluster PIN data). Due to time and space limitations, we are not able to perform computational experiments comparing FAC-PIN approach with those other six PIN clustering techniques; we leave this task as a future work. In [25], HC-PIN consistently outperforms those methods in terms of its (i) functional enrichment of the identified modules (ii) ability to detect both small-sized and large-sized modules, (iii) accuracies of the identified modules, (iv) ability to predict protein complexes, and (v) clustering efficiency. Both HC-PIN and CNM are currently the fastest agglomerative methods for clustering PIN data.

#### Functional enrichment validations

For the functional enrichment validations, we used DAVID's functional annotation tools http://david.abcc.ncifcrf.gov/[32] to identify enriched biological themes, particularly GO terms, and to estimate whether the predicted modules are biologically significant. DAVID uses a set of fuzzy classification algorithms to rank modules based on co-occurrences of their constituent proteins in annotation terms and computes a *P*-value indicating the significance of the module with respect to GO terms. The *P*-value is computed using an internal EASE score [33]. We used a *P*-value *cutoff *of 0.05 to find biologically significant clusters. A smaller *P*-value indicates that the predicted module is more biologically significant than one with a larger *P*-value

To estimate the performance of a network clustering algorithm in term of its ability to correctly identify the functional modules within a PIN, we also compute its *Recall*, *Precision*, and *F*-*Measure *as mapped to *C *as

(11) $$Recall=\frac{\left|C\cap F_{i}\right|}{\left|F_{i}\right|},$$

(12) $$Precision=\frac{\left|C\cap F_{i}\right|}{\left|C\right|},$$

(13) $$F-Measure=2\times \frac{Recall\times Precision}{Recall+Precision}$$

where, *C *is a module predicted by the algorithm, and *F_i _*is a known GO functional category mapped to *C *and considered as a *true predictions*. Thus, the proteins in *C ∩ F_i _*are the *true positive predictions*. *Recall *measures how effectively proteins with the same *F_i _*in the PIN are extracted, *Precision *measures how consistently proteins in the same *C *are annotated, and *F*-*Measure *is their harmonic mean [34]. The accuracy of the method is taken as the average *F*-*Measure *of the significant predicted modules. As in [25], we also only consider predicted modules of size 3 or more.

#### Protein complex validations

Protein complex validations proceed by determining the degree of overlap between the complexes identified by network clustering algorithm and the known protein complexes; that is, we want to determine how effectively an identified module matches a known complex. We used the *overlapping score *function given in [12,25,29,35]. The overlapping score, *O*(*C, K*), between a discovered complex *C *and a known complex *K *is defined as:

(14) $$O\left(C,K\right)=\frac{|C\cap K{|}^{2}}{\left|C|\times |K\right|},$$

in which a cluster *C *is considered to match a known complex *K *whenever *O*(*C, K*) ≥ *τ *; where, 0 < τ ≤ 1 is the matching threshold. We have a perfect match only when *O*(*C, K*) = 1. Threshold value *τ *= 0.2 was used in [12,25,35] whereas [29] used *τ *= 0.25. We used *τ *= 0.2 in our complex validation. After computing the overlapping scores between all pairs (*C, K*) of discovered complexes and known complexes for the PIN, we then determined the ability of the method to correctly classify the known complexes. The reason for doing this is that a given complex *K*_1 _may match many clusters but with different degrees of overlap, while another complex *K*_2 _may match with a single cluster only. Hence, we calculated the *Specificity*, the *Sensitivity*, and the *F*-*Score*, as our measures of accuracy here; they are defined as follows:

(15) $$Sensitivity=\frac{TP}{TP+FN},$$

(16) $$Specificity=\frac{TP}{TP+FP},$$

(17) $$F-Score=2\times \frac{specificity\times sensitivity}{specificity+sensitivity},$$

where, *TP *(true positive) is the number of the identified complexes *C *matched by the known complexes *K*, *FN *(false negative) is the number of known complexes that are not matched by the identified complexes, and *FP *(false positive) is the total number of the identified complexes *C *minus *TP*.

#### Modularity and efficiency analyses

All experiments in this paper were performed on an Intel machine (Core TM i7-2600, 3.400 GHz, CPU with 8 GB RAM). We compared FAC-PIN against HC-PIN and CNM in terms of the modularity of their clustering results and in terms of their computational efficiencies. For FAC-PIN, we ran it with its merging parameter set to *μ *= 0.5, then evaluated and reported the modularity of its resulting partition *P*_*k *_. The execution times (in seconds) are also recorded; the PINs are sorted in increasing order of their number of proteins *m*.

### Identification of functional modules in the *S. cerevisiae-2 *PINs

The computational results in this section are all generated with the merging parameter arbitrarily set to *μ *= 0.5 (except in Table 1) and with the modularity quality function *Q_w_*.

**Table 1:** The effect of variation of *μ *on clustering *S. cerevisiae-2 *PINs

*μ*	*k*	Max |*C_i_*|	Ave |*C_i_*|
0.25	203	265	21.498

0.5	232	374	18.810

1.0	413	155	10.567

1.5	489	120	8.924

1.75	491	111	8.888

#### Effect of the merging parameter *μ*

Table 1 shows the effect of parameter *μ *on FAC-PIN clustering results. Recall that a neighbor *u *of *v *is merged with the current cluster *C*(*v*) of *v *whenever the test

$$\begin{array}{c} R_{w}\left(u--\to v\right)>0.5\mu \\ \;\text{and}\\ R_{w}\left(u--\to v\right)\geq R_{w}\left(v--\to u\right) \end{array}$$

is satisfied for *u*. Hence, the size of a cluster *C*(*v*) increases as the merging parameter *μ *decreases since more neighbors are being merged together with *v*; and therefore, the number of clusters *k *also decreases as the sizes of clusters increase.

#### Functional enrichment of FAC-PIN modules

**Figure 1:**
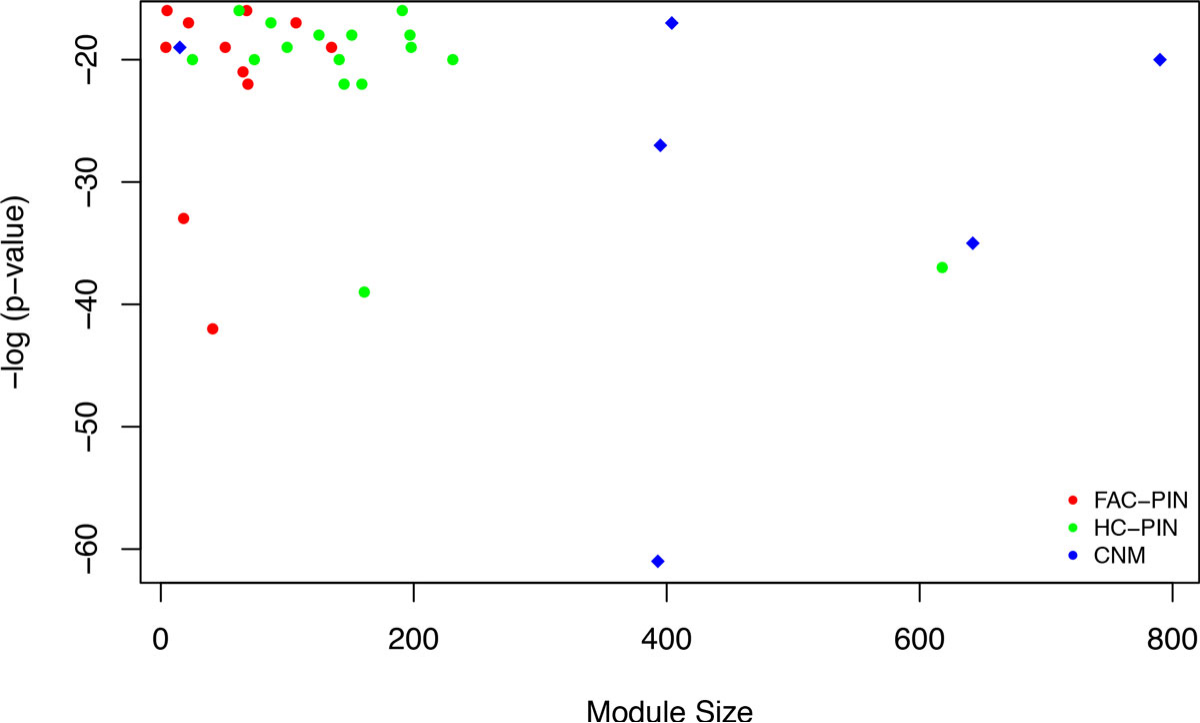
***P*-values versus Sizes of Modules**. Comparing sizes of enriched modules whose *P*-values fall in range [<E-15].

In Table 2, the three methods are compared for their functional enrichment of biological functions. The *P*- value from DAVID's internal EASE score is computed for each predicted module *C*, and a *P*-value *cutoff *of 0.05 is used to find the biologically significant clusters; a module whose *P*-value is above this cutoff is considered insignificant. The table shows, in this order, the number (percentage) and the average size of significant predicted modules with *P*-values falling within intervals: <E-15, [E-15, E-10], [E-10, E-5], and [E-5, 1]. Although CNM and HC-PIN show more enriched modules in the interval [<E-15], the modules with p-value falling in this range are much larger in CNM and HC-PIN than in FAC-PIN (specially CNM) with an average size of 439.83 for CNM and 103.1 for HC-PIN compared to 49.08 for FAC-PIN. Larger modules result in a high number of false positives, reducing the specificity of the highly-enriched modules. Figure 1 shows this trend. The figure compares the sizes of the modules whose enrichment *P*-values fall in the range [<E-15]. In the figure, there is a clear shift to the right in the case of CNM, indicating much larger modules. This trend is apparent in all *P*-values ranges (from Table 2). This indicates that CNM is the worst at predicting enrichment in small modules. HC-PIN's highly-enriched modules are also large compared to those produced by FAC-PIN, but their sizes are less than those of CNM. Also, FAC-PIN has the lowest rate of modules not passing the enrichment *P*-values cutoff of 0.05.

**Table 2:** Functional enrichment of the predicted modules which comprises of three or more *S. cerevisiae-2 *proteins; *μ *= 0.5

Algorithms	<E-15		[E-15, E-10]		[E-10, E-5]		[E-5, 1]	
	**N. Modules**	**Avg Size**	**N. Modules**	**Avg Size**	**N. Modules**	**Avg Size**	**N. Modules**	**Avg Size**

FAC-PIN	12 (8.1%)	49.08	18 (12.2%)	31.83	35 (23.6%)	25.57	73 (49.32%)	20.95

HC-PIN	16 (6.39%)	103.1	29 (23.77%)	63.23	38 (23.6%)	28.12	28 (22.95%)	25.11

CNM	6 (12.77%)	439.833	1 (2.1%)	71	5 (10.63%)	36.35	28 (59.58%)	28.89

#### Predicting large-sized versus small-sized modules

The *P*-value of a predicted module depends on its size, and hence, Table 3 and Table 4 show the accuracy of the methods respectively for predicting large and small modules.

**Table 3:** Performance comparison of the algorithms for predicting modules of size ≥ 20 on *S. cerevisiae-2 *PIN; *μ *= 0.5

Algorithms	Number of modules	Percentage of significant modules	Mean(-log *P*-value)	Mean(*F*-*Measure*)
FAC-PIN	58	98.28%	8.21	0.42

HC-PIN (*λ *= 1)	45	97.11%	12.25	0.31

CNM	17	96.43%	13.53	0.05

**Table 4:** Performance comparison of the algorithms for predicting modules of size ≤ 6 on *S. cerevisiae-2 *PIN; *μ *= 0.5

Algorithms	Number of modules	Percentage of significant modules	Mean(-log *P*-value)	Mean(*F*-*Measure*)
FAC-PIN	44	86.4%	6.16	0.41

HC-PIN (*λ *= 1)	33	59%	5.39	0.27

CNM	26	35.7%	1.81	0.08

In Table 3, we see that more than 96% of the modules predicted by each method are validated to be significant, though FAC-PIN yields a percentage slightly larger than that of HC-PIN or CNM. Although CNM gives the highest average -log *P-value*, it also yields the lowest average *F*-*measure*; this is due to the fact that its significant modules are much larger than those of HC-PIN and FAC-PIN, and hence, less accurate. FAC-PIN, on the other hand, predicted more accurate significant modules than HC-PIN and CNM but with the lowest average -log *P-value*; again, this is due to the smaller sizes of its generated modules.

In Table 4 however, performed consistently better than CNM and HC-PIN in all performance measures; FAC-PIN seems to be better at producing small-sized modules.

#### Accuracy of FAC-PIN

Table 5 lists the accuracy of each method with all the validations of *Biological Process *(BP), *Molecular Function *(MF), and *Cellular Component *(CC). Table 5 further confirms our analysis of the results in Table 3 and Table 4; that FAC-PIN predicts smaller but more accurate significant modules.

**Table 5:** Performance Comparison of the accuracy of FAC-PIN, HC-PIN, and CNM on *S. cerevisiae-2 *PIN; *μ *= 0.5

Accuracy for Modules of Size ≥ 3						
**Algorithms**	**Number of modules**	**Average size**	**Maximum size**	**Accuracy**		

				**BP**	**MF**	**CC**

FAC-PIN	148	28.24	374	0.42	0.30	0.65

HC-PIN (*λ *= 1)	122	43.19	483	0.39	0.28	0.52

CNM	47	88.59	790	0.22	0.23	0.25

**Accuracy for Modules of Size ≥ 2**						

FAC-PIN	232	18.8	374	0.39	0.32	0.57

HC-PIN (*λ *= 1)	172	23.74	483	0.37	0.30	0.44

CNM	147	29.68	790	0.09	0.15	0.21

### Identification of functional modules in the *S. cerevisiae-1 *PIN

Table 6 shows, in this order: the modularity value *Q_w_*(*P_k_*) of the generated partition *P_k_*; the number of predicted modules *k*_3 _with ≥ 3 proteins (and in parenthesis, the total *k*); and the average size $\bar{s}$ of the modules. Next, the validation results shows: the number *k_s _*of significant modules obtained overall (percentage of such modules is in parenthesis) and for each ontology class (*Biological Process*, *Cellular Component*, *Molecular Function*); the number of significant modules whose *P*-values fall within *P*-value interval <E-15, [E-15, E-10], [E-10, E-5], [E-5, 1] are listed next; the average $\bar{p}$ of -log *P*-value; and, the accuracy *A *of each algorithm as the average *F*-*Measure *of the predicted significant modules. The data set is the original unweighted PIN of *S. cerevisiae-1 *downloaded from the REACTOME database. In this PIN data, the number of modules discovered by FAC-PIN is comparable to (but still larger than) those detected by HC-PIN and CNM. FAC-PIN still predicts smaller and more accurate significant modules in this *S. cerevisiae-1 *with higher average -log *P-value*; which is consistent with our findings in the previous tables that FAC-PIN perform better due to the smaller sizes of its predicted modules.

**Table 6:** Functional enrichment of the predicted modules of un-weighted *S. cerevisiae-1 *PIN; *μ *= 0.5

Algorithms	*Q_w _*(*P_k _*)	*k*_3_	$\bar{s}$	Ontology	*k_s_*	<E-15	[E-15, E-10]	[E-10, E-5]	[E-5, 1]	$\bar{p}$	*A*
FAC-PIN	0.529	65 (90)	8.96	Overall	57 (63.33%)	2	7	23	25	4.21	0.137

				BP	24	2	1	11	10	4.09	0.165

				CC	18	0	4	6	8	4.21	0.123

				MF	15	0	2	6	7	4.01	0.096

HC-PIN	0.139	64 (87)	9.17	Overall	36 (42%)	7	5	12	12	3.17	0.024

				BP	10	2	3	3	2	3.02	0.028

				CC	14	3	2	4	5	2.97	0.032

				MF	12	2	0	5	5	3.15	0.029

CNM	0.248	61 (84)	9.62	Overall	19 (22%)	7	5	4	5	4.15	0.034

				BP	5	0	3	2	0	3.29	0.031

				CC	7	3	2	0	2	3.99	0.045

				MF	9	4	0	2	3	4.68	0.033

### Identification of protein complexes in the *S. cerevisiae-2 *PIN

Table 7 shows the *Specificity*, the *Sensitivity*, and the *F*-*Score *of the complexes identified by each method. The results are shown for the modularity scoring function *Q_w_*. For HC-PIN, results are shown for two values of its parameter *λ *as in [25]. The first three columns show, respectively, the number of proteins, the number of known complexes, and the average size of the known complexes in the data; columns 5, 6, and 7 are the number of discovered complexes, their average size, and the number of perfectly matched discovered complexes. In the table, we see that FAC-PIN discovers complexes whose average sizes (column 6) are closer to the average sizes of the known protein complexes (column 3), whereas HC-PIN and CNM predict farther average sizes. The consequence of this is that FAC-PIN complexes have higher accuracy in (*Specificity*, *Sensitivity *or *F*-*Score*). In particular, we obtain a larger number of perfectly matched complexes to communities with FAC-PIN than with HC-PIN or CNM.

**Table 7:** Comparison of the *Sensitivity*, *Specificity *and *F*-*Score *of FAC-PIN, HC-PIN and CNM

				Performances					
*P*	*K*	$\bar{S}$	Algorithms	*k*	$\left|\bar{k}\right|$	*k_m_*	*Sensitivity*	*Specificity*	*F*-*Score*
1318	144	9.153	FAC-PIN	158	8.35	9	0.61	0.54	0.592

			HC-PIN (*λ *= 0.5)	129	11.23	5	0.38	0.41	0.391

			HC-PIN (*λ *= 1.0)	117	12.83	3	0.29	0.32	0.31

			CNM	291	6.29	3	0.15	0.16	0.204

### Modularity and efficiency of FAC-PIN

Tables 8, 9, and 10 show the network modularity of the partitions obtained by the algorithms on the eight un-weighted PIN data downloaded from the REACTOME database, respectively for the modularity functions *Q_w_*, Ω*_w_*, and *D_w_*. The aim of both objectives *Q_w _*and Ω*_w _*is to optimize the modularity of the detected clusters (though Ω_*w *_yields clusters that are not too small and not too large, and therefore, it generates denser clusters than those from *Q_w_*); the aim of *D_w _*is to optimize both the modularity and the density of the clusters.

**Table 8:** Network modularity quality *Q_w _*results of FAC-PIN, HC-PIN, and CNM; *μ *= 0.5

Algorithms	Yeast	Finch Bird	Cattle	Wild Boar	Frog	Human	Zebra Fish	Rice
FAC-PIN	0.529	0.500	0.441	0.502	0.471	0.491	0.527	0.575

HC-PIN	0.139	0.498	0.418	0.419	0.319	0.218	0.198	0.529

CNM	0.248	0.766	0.693	0.626	0.754	0.719	0.736	0.348

**Table 9:** Network modularity quality Ω*_w _*results of FAC-PIN, HC-PIN, and CNM; *μ *= 0.5

Algorithm	Yeast	Finch Bird	Cattle	Wild Boar	Frog	Human	Zebra Fish	Rice
FAC-PIN	-1.370	-1.867	-1.704	-1.846	-1.839	-1.469	-1.825	-1.283

HC-PIN	-1.291	-0.131	-0.619	-0.948	-1.796	-0.823	-0.182	-1.279

CNM	-0.983	-1.315	-1.618	-1.848	-1.721	-1.441	-1.422	-0.819

**Table 10:** Network modularity density *D_w _*results of FAC-PIN, HC-PIN, and CNM; *μ *= 0.5

Algorithm	Yeast	Finch Bird	Cattle	Wild Boar	Frog	Human	Zebra Fish	Rice
FAC-PIN	77.534	164.501	149.350	164.501	149.003	152.540	136.916	101.841

HC-PIN	71.829	129.292	130.418	111.419	127.124	104.822	121.927	79.182

CNM	64.480	121.574	123.970	115.306	109.231	95.201	97.343	56.810

CNM is a modularity optimization algorithm designed to directly optimize the modularity quality function *Q_w_*, and hence, it is no surprise that it performed best with this function, as shown in Table 8. The modularity maximization process of CNM [23] yields a partitioning containing one very large cluster and many much smaller ones; this because, a node is selected to be included into the currently largest cluster first and to maximize the current *Q_w _*value. In the columns for *Rice *and *Yeast *in Table 8, we see that FAC-PIN outperforms CNM on *Q_w _*; Table 11 shows a possible reason for this, that the sizes max |*C_i_*| of their largest clusters are comparable.

**Table 11:** Comparing cluster statistics of FAC-PIN and CNM on *Q*_*w*_; *μ *= 0.5

Statistics	Algorithms	Yeast	Finch Bird	Cattle	Wild Boar	Frog	Human	Zebra Fish	Rice
*k*	FAC-PIN	90	247	285	267	268	269	379	154

	CNM	68	132	144	136	129	125	147	95

Ave |*C_i_*|	FAC-PIN	8.96	16.98	21.74	24.05	22.35	10.53	22.32	14.90

	CNM	10.47	32.33	43.94	47.93	47.13	48.70	58.57	24.46

Max |*C_i_*|	FAC-PIN	167	285	774	730	1043	1373	1104	541

	CNM	154	1199	1989	1471	2029	2029	2353	547

Recall that given a currently high-degree vertex *v *with its cluster *C_v_*, FAC-PIN merges it with all its neighbors *u *satisfying the merging condition

$$\begin{array}{c} R_{w}\left(u--\to v\right)>0.5\mu \\ \;\text{and}\\ R_{w}\left(u--\to v\right)\geq R_{w}\left(v--\to u\right). \end{array}$$

The first term in the merging condition guarantees that only edges (*u, v*) which have low local betweenness value $\lambda \left(u,v\right)=100\cdot \frac{1}{|N_{u}\cap N_{v}|+1}$ are considered for possible inclusion in the induced subgraph *C*(*v*) of *C_v_*. The second term guarantees that only those neighbors *u *which can contribute more edges to *C*(*v*), than *v *contributes to *C*(*u*), are selected. Hence, FAC-PIN merges neighbors *u *which contribute low local betweenness edges while optimizing the density of *C*(*v*). Also as said before, the relative vertex clustering value $R_{w}(u--\to v)$ combines the principles behind the vertex clustering coefficient of [14], the edge clustering coefficient $C_{u,v}^{\left(k\right)}$ of [2], and the edge clustering value *ECV *(*u, v*) of [25]. Since the objectives of Ω*_w _*and *D_w _*is to seek for modular partitioning containing dense clusters, we can see that in both Tables 9 and 10, FAC-PIN outperformed both HC-PIN and CNM on both modularity function Ω*_w_*; in seven out of eight PIN data for Ω*_w_*, and in all PIN data for *D_w_*. In particular for *D_w_*, FAC-PIN yield much higher modularity values.

Table 12 shows the execution times (in seconds) of each algorithm and the same data sets as above, but for modularity function *Q_w _*only. As one can see, FAC-PIN ran faster than both HC-PIN and CNM on all data sets.

**Table 12:** Execution times of FAC-PIN, HC-PIN, and CNM; using *Q_w _*and *μ *= 0.5

PINs	Number of Proteins	Number of Interactions	FAC-PIN	HC-PIN	CNM
Yeast	5697	40675	313.315	446.231	501.239

Finch Bird	3929	74314	235.804	610.238	441.365

Cattle	5737	113888	300.766	781.231	596.833

Wild Boar	5303	119920	649.483	691.472	972.213

Frog	5473	122706	429.873	1021.432	912.692

Human	12994	135935	533.000	702.325	822.511

Zebra Fish	8188	274358	874.303	1183.350	1238.281

Rice	3778	320570	349.712	539.329	1281.273

## Conclusions

In this paper, we have proposed a new agglomerative clustering approach, FAC-PIN algorithm, for detecting the communities of a given PIN networks, and then compared our method with two fast hierarchical techniques discussed in literature. Our approach is based on the use of a new measure, the *relative vertex-to-vertex clustering value *which helps decide whether a given vertex *u *should be included within the cluster of another vertex *v *depending on how many of its neighbors form a triangle with *v*. Our approach is very fast since we are clustering vertices not edges, as in the compared methods. Thus our method is appropriate for PIN data, which in general contain more interactions than proteins. More study needs to be done, in particular the validation based on random networks, in order to analyze the robustness of FAC-PIN. Comparisons with other methods which are not necessarily hierarchical will also be important. Non-agglomerative clustering methods based on the relative vertex-to-vertex clustering value will be investigated. In this current version of FAC-PIN, a neighbor *u *is merged with a cluster $C_{v_{i}}$ whenever its $R_{w}(u--\to v_{i})$ value satisfies the merging condition and irrespective of whether there is another vertex *vj *such that $R_{w}(u--\to v_{j})$ also satisfies the condition; we, therefore, plan a new variant of FAC-PIN in which each node *u selects *the *best *neighbor *v *to be merged with. Finally, we plan to modify FAC-PIN for directed (un-weighted and weighted) protein interaction networks.

As a final note: we have not made experiments on weighted PINs. In our initial submission, we have used the following weighted criterium:

$$R_{w}\left(u--\to v\right)=\frac{\sum_{a\in I_{u,v}^{+};\left(u,a\right)\in E}w\left(u,a\right)}{\sum_{b\in N_{u}^{+};\left(u,b\right)\in E}w\left(u,b\right)}$$

One of the reviewer of the initial manuscript has pointed out that this formula is incorrect since it depends only on the weights of edges connected to node *u*, not of the edges connected to *v*. An important consequence of this error, is that our analysis of $R_{w}(u--\to v)$ (based on the formula above) will apply to the unweighted case only but will not necessarily apply to the weighted case. We have verified this, both computationally and theoretically, before engaging to experiment on weighted PINs. Due to time constraint, it is now impossible to perform and complete the experiments on weighted PINs using the correct formula in Equation (10). Our plan for the immediate future is therefore to perform these experiments.

## Competing interests

The authors declare that they have no competing interests.

## Authors' contributions

AN proposed the current forms of the *Relative Vertex-to-Vertex Clustering Value*, introduced an initial version of the FAC-PIN algorithm, and suggested the experiments to be performed. ZMI proposed and implemented the current version of the FAC-PIN algorithm, and performed all the suggested computational experiments. AN and ZMI have equally contributed in writing the paper.

## Declarations

The publication of this article is funded by the National Science and Engineering Council of Canada (NSERC).

AN declares that he was not invovled in the peer review process or any acceptance decisions regarding this article on which he is an author.

This article has been published as part of *BMC Bioinformatics *Volume 16 Supplement 4, 2015: Selected articles from the 9th IAPR conference on Pattern Recognition in Bioinformatics. The full contents of the supplement are available online at http://www.biomedcentral.com/bmcbioinformatics/supplements/16/S4.
